# Efficient induction of functional ameloblasts from human keratinocyte stem cells

**DOI:** 10.1186/s13287-018-0822-4

**Published:** 2018-05-02

**Authors:** Xuefeng Hu, Jyh-Wei Lee, Xi Zheng, Junhua Zhang, Xin Lin, Yingnan Song, Bingmei Wang, Xiaoxiao Hu, Hao-Hueng Chang, Yiping Chen, Chun-Pin Lin, Yanding Zhang

**Affiliations:** 1 Southern Center for Biomedical Research, Fujian Normal University, Fuzhou, 350108 China; 2 Fujian Key Laboratory of Developmental and Neural Biology, College of Life Science, Fujian Normal University, Fuzhou, 350108 China; 3 Department of Materials Engineering, Ming Chi University of Technology, New Taipei, 24301 Taiwan; 4 Center for Thin Film Technologies and Applications, Ming Chi University of Technology, New Taipei, 24301 Taiwan; 5 College of Engineering, Chang Gung University, Taoyuan, 33302 Taiwan; 6 School of Dentistry, National Taiwan University and National Taiwan University Hospital, Taipei, 10048 Taiwan; 7 Department of Cell and Molecular Biology, Tulane University, New Orleans, LA 70118 USA; 8 Graduate Institute of Clinical Dentistry, School of Dentistry, National Taiwan University and National Taiwan University Hospital, Taipei, 10048 Taiwan

**Keywords:** Fibroblast growth factor 8, Sonic hedgehog, Keratinocyte stem cells, Ameloblast, Enamel

## Abstract

**Background:**

Although adult human tissue-derived epidermal stem cells are capable of differentiating into enamel-secreting ameloblasts and forming teeth with regenerated enamel when recombined with mouse dental mesenchyme that possesses odontogenic potential, the induction rate is relatively low. In addition, whether the regenerated enamel retains a running pattern of prism identical to and acquires mechanical properties comparable with human enamel indeed warrants further study.

**Methods:**

Cultured human keratinocyte stem cells (hKSCs) were treated with fibroblast growth factor 8 (FGF8) and Sonic hedgehog (SHH) for 18 h or 36 h prior to being recombined with E13.5 mouse dental mesenchyme with implantation of FGF8 and SHH-soaked agarose beads into reconstructed chimeric tooth germs. Recombinant tooth germs were subjected to kidney capsule culture in nude mice. Harvested samples at various time points were processed for histological, immunohistochemical, *TUNEL*, and western blot analysis. Scanning electronic microscopy and a nanoindentation test were further employed to analyze the prism running pattern and mechanical properties of the regenerated enamel.

**Results:**

Treatment of hKSCs with both FGF8 and SHH prior to tissue recombination greatly enhanced the rate of tooth-like structure formation to about 70%. FGF8 and SHH dramatically enhanced stemness of cultured hKSCs. Scanning electron microscopic analysis revealed the running pattern of intact prisms of regenerated enamel is similar to that of human enamel. The nanoindentation test indicated that, although much softer than human child and adult mouse enamel, mechanical properties of the regenerated enamel improved as the culture time was extended.

**Conclusions:**

Application of FGF8 and SHH proteins in cultured hKSCs improves stemness but does not facilitate odontogenic fate of hKSCs, resulting in an enhanced efficiency of ameloblastic differentiation of hKSCs and tooth formation in human–mouse chimeric tooth germs.

**Electronic supplementary material:**

The online version of this article (10.1186/s13287-018-0822-4) contains supplementary material, which is available to authorized users.

## Background

Various efforts to develop techniques for human tooth regenerative therapy and replacement have been attempted for decades [1, 2]. Currently, bioengineering of a whole tooth crown from embryonic tooth germ cells appears to be the most successful approach for tooth regeneration in several animal models including mouse, rat, pig, and dog [3–10]. Impressively, it was reported that implantation of a bioengineered tooth germ, reconstructed from mouse embryonic dental epithelial and mesenchymal cells, into a lost tooth socket in the alveolar bone of adult mice could develop into a fully functional tooth [11–14], indicating the feasibility of future regenerative therapy in humans via implantation of bioengineered tooth germs. However, in practice, it is impossible to use embryonic cells for such clinical therapy. Thus, identification of adult cell sources, such as stem cells from adult tissue or induced pluripotent stem cells (iPSCs), for *ex-vivo* generation of implantable tooth germ is a prerequisite for the realization of human biotooth replacement therapy in the future.

Stem cell-based tissue engineering has been proven a prospective approach to repair or replace an injured tissue or organ. Adult bone marrow stem cells (bone marrow stromal cells) are the first adult cell source capable of participating in tooth formation when confronted with the mouse embryonic dental epithelium that possesses odontogenic inducing capability [15]. At least five types of mesenchymal stem cells from adult human teeth have been isolated [16]. Among them, dental pulp stem cells (DPSCs), stem cells from exfoliated deciduous teeth (SHED), and stem cells from the apical papilla (SCAP) could generate dentin/pulp-like complexes in *ex-vivo* culture [17–19]. Although these adult dental stem cells do not possess either odontogenic inducing capability or competence to support tooth formation when confronted with embryonic dental epithelia [20], they remain promising stem cell sources for regeneration of tooth mesenchymal components. On the other hand, the postnatal dental epithelium-derived stem cells are more difficult to obtain due to ameloblastic apoptosis during tooth eruption. It was reported that subcultured epithelial cell rests of Malassez can differentiate into ameloblast-like cells and generate enamel-like tissues in combination with dental pulp cells at the crown formation stage [21]. We and others have reported previously that nondental epithelia-derived human stem cells including human keratinocyte stem cells (hKSCs) [20, 22], gingival epithelial cells [23], and iPSCs [24], when recombined with either human or mouse embryonic dental mesenchyme, could support tooth formation and differentiate into enamel-secreting ameloblasts. However, less than 30% and 10% of these recombinant explants in subrenal culture formed teeth and produced enamel, respectively [22]. Such low efficiency of ameloblastic differentiation prevents use of these human stem cells as realistic cell sources for tooth replacement therapy. In addition, whether hKSC-derived dental epithelia exhibit an unusual life cycle and whether the regenerated enamel acquires the unique physicochemical characteristics remain elusive and warrant further exploration.

Studies indicated that either FGF8 or SHH alone is sufficient to promote limb regeneration in amphibian [25]. FGF8 or SHH is able to stimulate neurite outgrowth and cavernous nerve regeneration in vitro, respectively [26, 27]. In the tooth, FGF8 promotes cell proliferation and inhibits apoptosis in diastemal tooth epithelium, and revitalizes the tooth developmental program [28]. In this study, we developed an approach that greatly enhanced the ratio of ameloblastic differentiation of hKSCs and formation of tooth-like structures in tissue recombinants. We further examined the developmental process of differentiation of the hKSC-derived dental epithelium and present evidence for rapid differentiation of human ameloblasts and production of regenerated enamel with intact prisms the same as normal enamel. Meanwhile, we observed an increasing tendency for mineralization effect with improved mechanical properties in the regenerated enamel as cultivation extends. Our results provide a significant advance toward future use of human adult stem cells to generate implantable tooth organ *ex vivo* by tissue-engineering approaches.

## Methods

### Culture of hKSCs and application of recombinant proteins

Circumcised human foreskins from children 5–12 years old were collected immediately after surgery from Fuzhou Children's Hospital in Fujian Province. Primary human keratinocytes were isolated and cultivated in Keratinocyte Serum-free Medium (KSFM; Gibco) according to the protocol described previously [22]. Keratinocyte stem cells were characterized by cell surface markers as described previously [22]. Recombinant human FGF8a (100 ng/ml; R&D Systems) and/or SHH (100 ng/ml; R&D Systems) proteins were applied to passage 3 hKSCs cultured in KSFM at 90% confluence in 10-cm culture dishes. These cells were continuously cultured for 18 h or 36 h prior to being used for subsequent tissue recombinant experiments or immunocytochemical assay.

### Tissue recombination and subrenal capsule culture

Tissue recombination and mouse subrenal culture were carried out as described previously [22]. Briefly, mandibular molar tooth germs dissected from E13.5 mouse embryos were incubated in 2.25% trypsin and 0.75% pancreatin in PBS on ice for 10 min and then dental epithelia were removed with fine forceps. Pieces of confluent hKSC sheets were recombined with E13.5 mouse dental mesenchyme to reconstruct human–mouse chimeric tooth germs [22]. Agarose beads (Bio-Rad) soaked with FGF8 (125 ng/μl; R&D Systems) and/or SHH (250 ng/μl; R&D Systems), respectively, were implanted into tissue recombinants as described previously [22]. BSA beads were used as negative control. Recombinant tooth germs were cultured in Trowell type organ culture for 24 h prior to being subjected to subrenal culture in immune-compromised adult male mice. Samples were harvested at various time points after subrenal culture and processed for histological analysis and immunohistochemical staining.

### Histology, immunochemical staining, TUNEL assay, and western blot analysis

Molar tooth germs dissected from surgically terminated human fetuses of 12th-week, 16th-week, and 19th-week gestation were provided by Fujian Province Maternal and Child Health Hospital. Use of human embryonic tissues in this study was approved by the Ethics Committee of Fujian Normal University, Fuzhou, China, and use of animals was approved by the Animal Use Committee of Fujian Normal University. Human fetus tooth germs and harvested recombinant samples treated with FGF8 and SHH protein prior to tissue recombination were fixed in 4% paraformaldehyde (PFA) overnight at 4 °C on a rotator. Calcified tissues were further decalcified in 10% ethylenediaminetetraacetic acid (EDTA) for 1 week prior to being processed for dehydration and paraffin embedding. Sections were made at 10 μm, and were subjected to hematoxylin/eosin staining or Azan dichromic staining for histological analysis, and to immunohistochemical staining by antigen recovery technique. The following antibodies were used: anti-human ameloblastin, anti-human K18, anti-human p63, anti-human K10, anti-human integrin-β1 (Santa Cruz Biotech, Inc.), anti-human amelogenin, anti-human Sp3 (Abcam), anti-human Sp6, and anti-human Msx2 (HPA). For negative controls, the primary antibodies were omitted. Immunostaining, immunofluorescence, and TUNEL assay (Roche) procedures followed the instructions of the manufacturers. For western blot analysis, cultured hKSCs were extracted with urea lysis buffer. Equal amounts of samples were electrophoresed on 12% SDS polyacrylamide gels and transferred to NC membrane (Millipore). Immunoreactions were performed with the specific primary antibodies as mentioned earlier, visualized with fluorescent secondary antibodies (LI-COR), and scanned on an Odyssey Clx Imager (LI-COR). Blot images were quantified by densitometric analysis with ImageJ software.

### Scanning electronic microscopy

The surface morphologies of human tooth (adult and child), mouse molar, and human–mouse chimeric tooth crown specimens that were treated with both FGF8 and SHH were investigated using a scanning electron microscope (S-3400 N; Hitachi, Japan) with an acceleration voltage of 15 kV. Each specimen was cold mounted in resin, abraded with #1200 SiC paper, polished with 0.05 μm alumina powder, etched with 25% EDTA for 60 s, washed in distilled water, and ultrasonically degreased in acetone. The conductive Pt thin film around 5 nm thick was sputtered on each specimen before scanning electronic microscopy (SEM) analysis.

### Nanoindentation test

The nanoindentation hardness, *H*, and elastic modulus, *E*, of human (adult and child), mouse molar, and human–mouse chimeric tooth crown specimens that were treated with FGF8 and SHH were investigated by means of a nanoindenter (TI-900, TriboIndenter; Hysitron, USA) with a Berkovich 142.3° diamond probe at different loads to achieve a fixed indentation depth of 70 nm. The loading rates were between 6 and 14 μN/s. Before the nanoindentation test, each specimen was cold mounted in resin, abraded with #1200 SiC paper, polished with 0.05 μm alumina powder, washed in distilled water, and ultrasonically degreased in acetone. Ten indentation tests were performed on the enamel and dentin regions, respectively, for each specimen. The hardness and elastic modulus of each indent were determined on the basis of the Oliver and Pharr method [29]. The elastic modulus, *E*, was expressed as follows:

$$ \frac{1}{Er}=\frac{1-{\nu}^2}{E}+\frac{1-{\nu_i}^2}{E_i}, $$

where *E*_*r*_ and *ν* are the reduced elastic modulus and Poisson’s ratio, respectively, for the specimen under test, and *E*_*i*_ (1140GPa) and *ν*_*i*_ (0.07) are the corresponding parameters of the diamond indenter. The Poisson ratio, *ν*, was 0.3 for each tooth sample [30]. The fused quartz standard sample was used to calibrate the area function of the nanoindenter [29].

## Results

### Enhanced ameloblastic differentiation efficiency of cultured hKSCs in the presence of FGF8 and SHH

Although our previous studies manifested that hKSCs, when recombined with E13.5 mouse dental mesenchyme, were induced to differentiate into enamel-secreting ameloblasts in the presence of FGF8-asorbed beads, the efficiency was quite low with hKSCs differentiating to ameloblasts in 13 out of 41 formed teeth from 146 tissue recombinants [22]. Enhancement of the ameloblastic differentiation rate indeed warranted further exploration. SHH represents one of the pivotal cell-autonomous factors expressed in the developing human and mouse dental epithelium, being required for the development of early tooth germ as well as involved in the determination of ameloblastic cytodifferentiation and function [31]. This prompted us to investigate whether application of FGF8/SHH-absorbed beads, instead of FGF8-absorbed beads alone, in the human–mouse chimeric tooth germ could increase the efficiency of tooth formation and ameloblastic differentiation. Indeed, our tissue recombination experiments showed that 12 out of 25 recombinant samples developed into chimeric teeth, with enamel deposition in eight of these cases (Table 1). The efficiency increased to around 50% for tooth formation and 65% for ameloblastic differentiation in formed tooth-like structures. These promising results again encouraged us to test whether application of FGF8 and SHH proteins in the cultured hKSCs prior to tissue recombination could further increase the ratio of tooth formation and ameloblastic differentiation in the tissue recombinants.

**Table 1:** Success ratio of tooth formation and ameloblastic differentiation in tissue recombinants

Protein in culture (h)		Protein beads in recombinant		Number of recombinants	Number of tooth formations	Ratio of tooth formation (%)	Number of ameloblastic differentiations	Ratio of ameloblastic differentiation (%)
FGF8	SHH	FGF8	SHH					
–	–	+	+	25	12	48.0	8	66.7
18	–	+	+	11	5	45.5	5	100
–	18	–	–	10	1	10.0	1	100
18	18	+	+	17	9	52.9	9	100
36	36	+	+	13	9	69.2	9	100

*FGF8* fibroblast growth factor 8, *SHH* Sonic hedgehog

We next treated the cultured hKSCs with either FGF8 (100 ng/ml) or SHH (100 ng/ml) proteins, or both of them, for 18 h or 36 h before proceeding to tissue recombination and subrenal culture (Table 1). It is noteworthy that hKSCs treated with either FGF8 or SHH alone, or both of them, respectively, retain much more healthy morphology with a smaller and typical cobblestone-like cell shape by comparison with control cells treated with PBS exhibiting a bigger and flattened phenotype (data not shown). Histological examination revealed that in samples cultured for 18 h, although the ratio of tooth formation remained around 50% (5 out of 11 recombinant samples) in the presence of FGF8 protein alone or in the presence of both FGF8 and SHH (9 out 17 recombinants), all teeth formed in both conditions exhibited a 100% ratio of ameloblastic differentiation (Table 1). We then extended the protein-treated culture duration to 36 h, and the tooth forming rate increased to 69% (9/13) with enamel deposition in all cases of the tooth forming samples (Table 1). However, treatment of cultured hKSCs with FGF8/SHH for periods longer than 36 h (48 and 60 h) resulted in decreased tooth formation and reduced ameloblastic differentiation in tissue recombinants (data not shown). Our results, therefore, definitely demonstrated a dramatically enhanced efficiency of ameloblastic differentiation and tooth formation by application of FGF8 and SHH protein in construction of human–mouse chimeric tooth recombinants.

### FGF8 and SHH improves stemness but not ameloblastic fate of cultured hKSCs

**Fig. 1:**
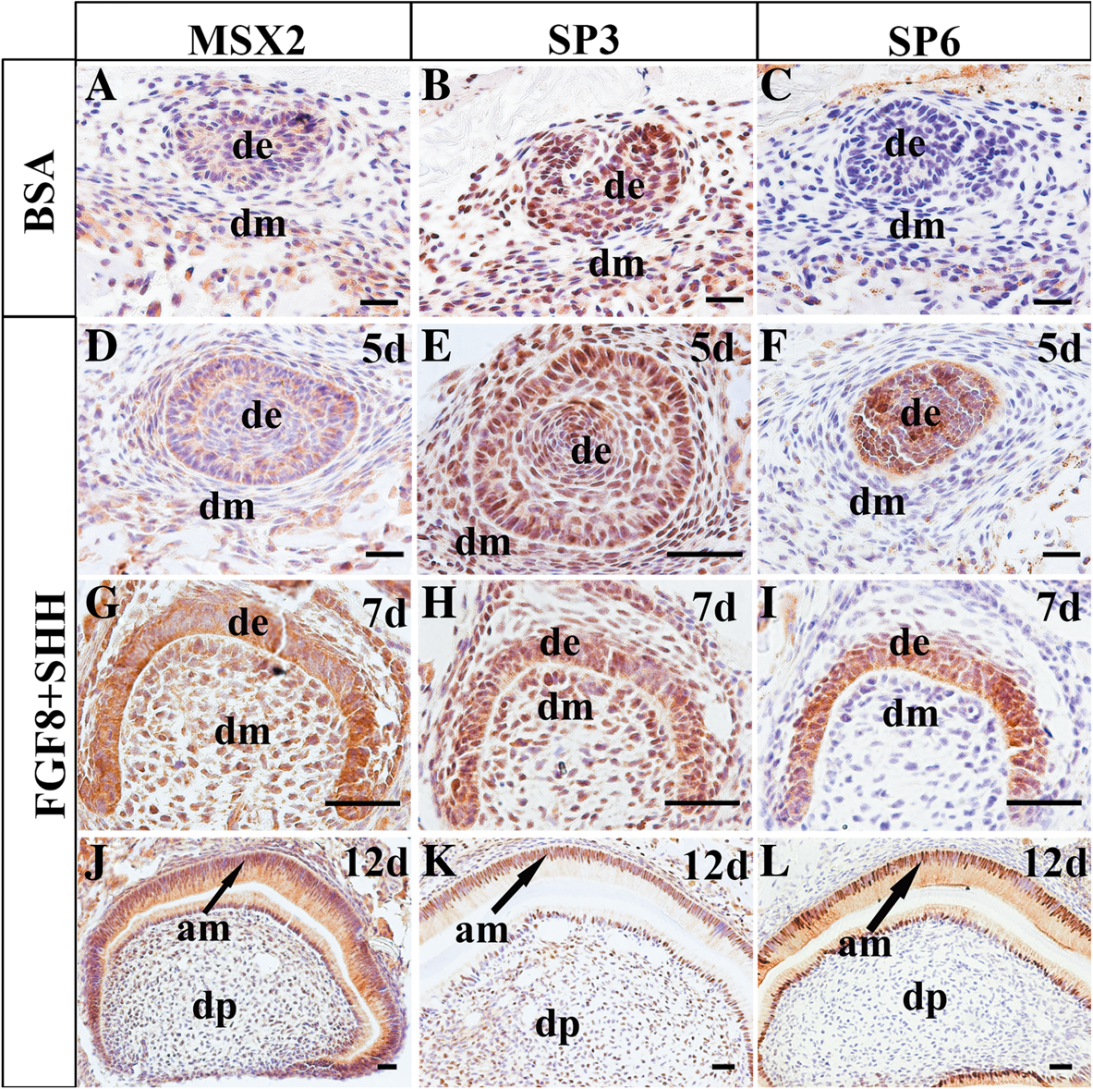
Immunohistochemical examination of MSX2, SP3, and SP6 expression in human–mouse chimeric teeth in presence or absence of FGF8 and SHH. **A**–**C** Expression of MSX2, SP3, and SP6 in BSA-treated control samples after 5 days in subrenal culture. MSX2 (**A**) and SP3 (**B**), but not SP6 (**C**), are detected in recombinant implants. **D–I** Expression of MSX2, SP3, and SP6 in human–mouse chimeric tooth germs in presence of FGF8 and SHH at various time points after subrenal culture. MSX2 and SP3 proteins detected in dental epithelium and mesenchyme, while SP6 exclusively present in dental epithelium at 5 days (**D–F**) and 7 days (**F–I**) after grafting. All three proteins present in both ameloblasts and odontoblasts at 12 days after grafting (**J–l**). Scale bar = 50 μm. BSA bovine serum albumin, FGF8 fibroblast growth factor 8, SHH Sonic hedgehog, 5d 5 days, de dental epithelium, dm dental mesenchyme, am ameloblast, dp dental pulp

**Fig. 2:**
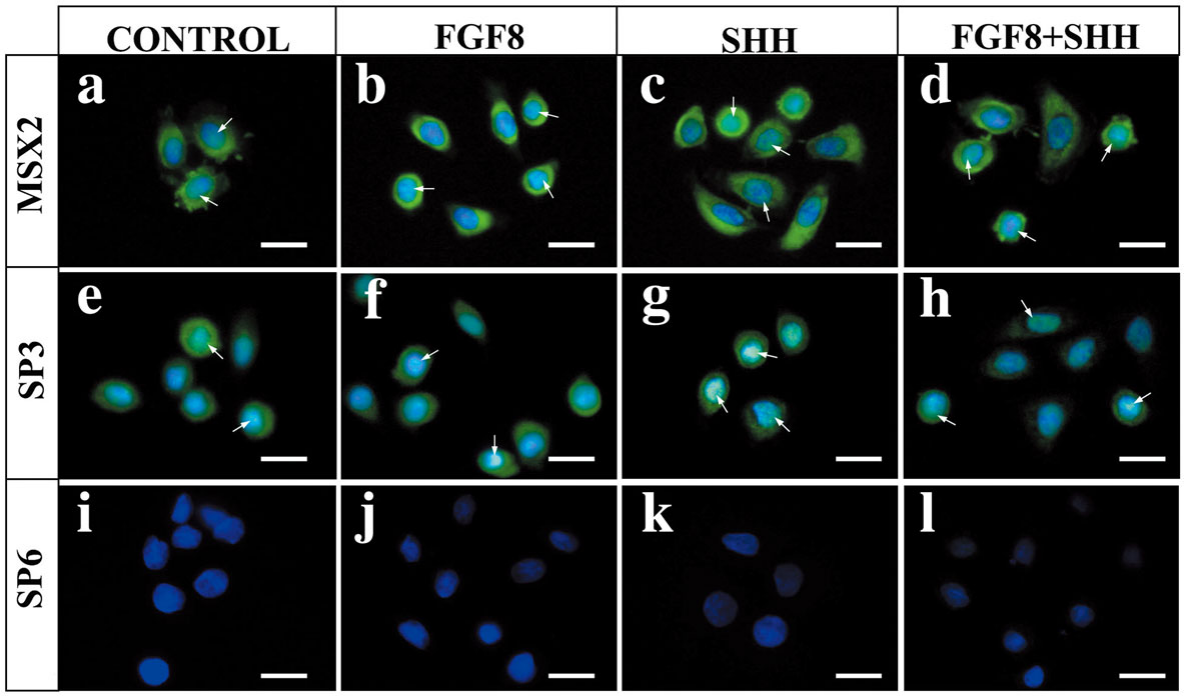
Expression of MSX2, SP3, and SP6 in cultured hKSCs in presence and/or absence of FGF8 and/or SHH. hKSCs at passage 3 cultured with or without proteins for 36 h prior to immunofluorescent staining. No expression differences found among controls (**a**, **e**, **i**) and experimental groups treated with FGF8 (**b**, **f**, **j**), SHH (**c**, **g**, **k**), or FGF8 + SHH (**d**, **h**, **l**). Scale bar = 100 μm. FGF8 fibroblast growth factor 8, SHH Sonic hedgehog

Next, we studied why addition of FGF8 and SHH proteins in the cultured hKSCs or in human–mouse tissue recombinants could dramatically increase the efficiency of tooth formation and ameloblastic differentiation. The transcription factors *Msx2* [32, 33], *Sp3* [34], and *Sp6* [35] have been demonstrated to be involved in ameloblastic differentiation in mice. Mutations of each of these genes in mice or humans disrupted ameloblastic differentiation and resulted in amelogenesis imperfecta. To unveil whether these transcription factors were involved in ameloblastic differentiation of hKSCs in the tissue recombinant in the presence or absence of FGF8 and SHH protein, we first carried out immunostaining to confirm whether MSX2, SP3, and SP6 were expressed in human tooth germs. We did find that these proteins were all present in the dental epithelium and differentiating ameloblasts in human deciduous tooth germs (Additional file 1: Figure S1), exhibiting identical expression patterns to those in mice [32–36]. We then examined the expression of MSX2, SP3, and SP6 in the hKSC-derived dental epithelium in human–mouse chimeric tooth germs after 5-day subrenal culture. We found that MSX2 and SP3, but not SP6, proteins were presented in BSA control samples, which were neither treated with FGF8 and SHH proteins in cultured hKSCs nor implanted with FGF8/SHH-soaked beads (Fig. 1a–c). In comparison, however, in addition to the expression of MSX2 and SP3, SP6 was strongly activated in the dental epithelium (Fig. 1d–f) of chimeric teeth with well-differentiated preameloblasts (Fig. 1g–i) and enamel-secreting ameloblasts (Fig. 1j–l) in the FGF8/SHH-treated samples. Furthermore, unlike MSX2 and SP3 that exhibited more widely spread expression patterns, SP6 showed an exclusive expression pattern as in normal human tooth development in the inner enamel epithelium (Additional file 1: Figure S1), the dental epithelium that directly differentiates into functional ameloblasts, in the chimeric recombinants. Considering the presence of MSX2 and SP3 proteins in the cultured KSCs (Fig. 2), these findings implied that the activation of SP6 in the hKSC-derived epithelium by mouse embryonic dental mesenchyme in the presence of FGF8 and SHH may be necessary for ameloblastic differentiation in the chimeric tooth.

Since application of FGF8 and SHH proteins in cultured hKSCs could dramatically increase the efficiency of ameloblastic differentiation and tooth formation in the human–mouse chimeric tooth germ, we investigated whether application of these proteins would commit cultured hKSCs to the odontogenic fate through induction of MSX2, SP3, and SP6 expression. We examined effects of FGF8 and SHH proteins on the expression of these three transcription factors in cultured hKSCs using immunofluorescence. Our results revealed that in control cells MSX2 and SP3 proteins were detectable whereas SP6 was not detectable (Fig. 2a–i). However, expression profiles of SP6, as well as MSX2 and SP3, remained unchanged in cultured hKSCs that were treated either with FGF8 (Fig. 2b, f, j) or SHH (Fig. 2c, g, k) alone or as a combination (Fig. 2d, h, l) for 18 h or 36 h. These results suggested that enhanced efficiency of ameloblastic differentiation and tooth formation by application of FGF8 and SHH in cultured hKSCs prior to tissue recombination might not be associated with commitment of hKSCs to odontogenic fate by activation of transcription factors of odontogenic importance.

**Fig. 3:**
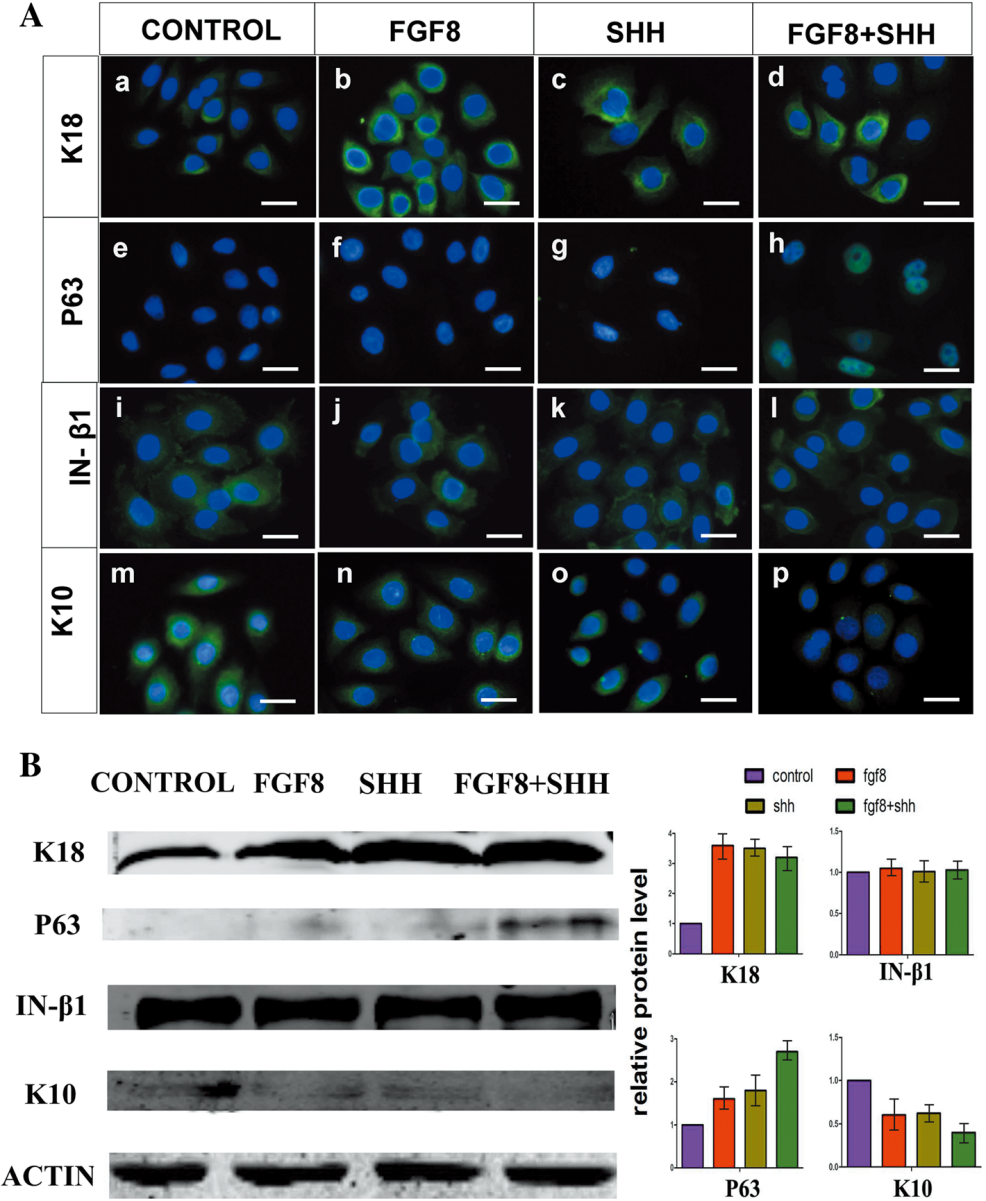
Enhanced stemness of hKSCs in presence of FGF8 and/or SHH. **A** Immunofluorescence shows increased expression levels of K18 (a–d) and p63 (e–h), unaltered expression of integrin-β1 (i–l), and decreased K10 expression (m–p) in cultured hKSCs in the presence of FGF8 and/or SHH. **B** Western blot and densitometric quantification analyses further confirm results of immunofluorescence. Scale bar = 100 μm. FGF8 fibroblast growth factor 8, SHH Sonic hedgehog, IN integrin

Stemness is a critical element for differentiation capability of stem cells. To illustrate possible roles of FGF8 and SHH for improving ameloblastic differentiation of cultured hKSCs, we further investigated expression patterns of stemness markers of epidermal stem cells after growth factor treatment, including K18, p63, integrin-β1, and K10 [37]. Figure 3 shows immunocytochemical and western blot analyses of the expression profile of cultured hKSC stemness markers. Our results indicated that, compared to the controls that expressed a very low level of K18, application of FGF8 or SHH protein alone or both almost doubled the expression level of K18 in cultured hKSCs (Fig. 3Aa–d, B). In addition, nuclear p63, a key stemness marker of epidermal stem cells, was barely detectable in BSA-treated controls while its expression level was increased slightly in cultured hKSCs with either FGF8 or SHH treatment (Fig. 3Ae–g, B). On the contrary, p63 was abundantly expressed in cultured hKSCs with treatment by both FGF8 and SHH together (Fig. 3Ah, B). The expression level of K10, a differentiating marker of epidermal stem cells, declined in these cultured cells, exhibiting a reversed pattern as compared to p63 and K18 after growth factor treatment (Fig. 3Am–p, B). Moreover, we observed no difference in expression level of integrin-β1, another marker of epidermal stem cells, among the different experimental groups (Fig. 3Ai–l, B). These findings strongly suggested that application of FGF8 and SHH protein in cultured hKSCs improves stemness but does not facilitate odontogenic fate of hKSCs, resulting in an enhanced efficiency of ameloblastic differentiation of hKSCs and tooth formation in human–mouse chimeric teeth. In addition, due to fast decaying of active FGF8 and SHH in the cell culture, analysis of hKSC cultured for 48 and 60 h revealed an obvious decrement of stemness (data not shown), further supporting the earlier idea.

### The developmental process of differentiation of hKSC-derived dental epithelium into functional ameloblasts

**Fig. 4:**
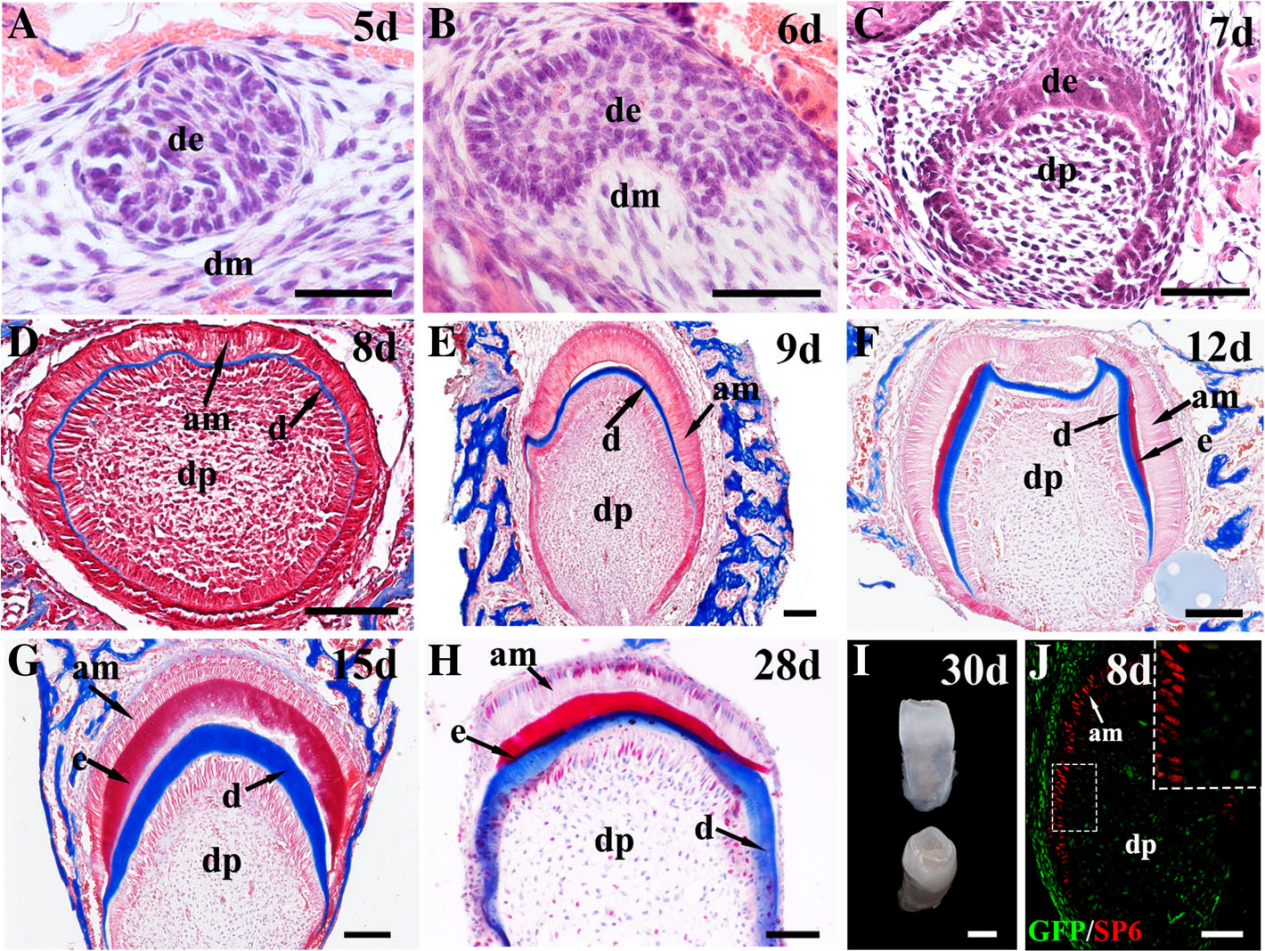
Histogenesis of hKSC-derived dental epithelium in human–mouse chimeric teeth. Sections through chimeric teeth retrieved from subrenal culture at different time points processed with hematoxylin and eosin (**A–C**) or Azan dichromic staining that stains dentin (d) blue and enamel (e) red (**D–H**). **a** hKSC-derived epithelial bud (de) formed after 5 days in subrenal culture. **B–E** Tooth buds underwent typical dental epithelial histogenesis, forming the cap-like structure at day 6 (**B**), the bell-like structure at day 7 (**C**), elongated preameloblasts at day 8 (**D**), and well-differentiated ameloblasts (am) at day 9 (**E**), respectively, after subrenal culture. **F** hKSC-derived ameloblasts began to deposit enamel (e) on the surface of dentin (d) around day 12 after subrenal culture. **G** Thick layer of enamel secreted from hKSC-derived ameloblasts at around day 15 after subrenal culture. **H** Reduced thickness and compacted layer of enamel found in a graft cultured for 28 days. **I** A lateral (left) and top (right) view of human-mouse chimeric tooth crowns formed after 30 days in subrenal culture. **J** hKSC-derived ameloblasts expressed human SP6 but not GFP , and the odontoblasts and dental pulp cells of eGFP-mouse origin expressed GFP in human-mouse chimeric tooth crown after 8 days culture. stain Scale bar = 100µm (**A-H**, **J**), 500µm (**I**). 5d 5 days, dm dental mesenchyme, dp dental pulp, GFP green fluorescent protein

**Fig. 5:**
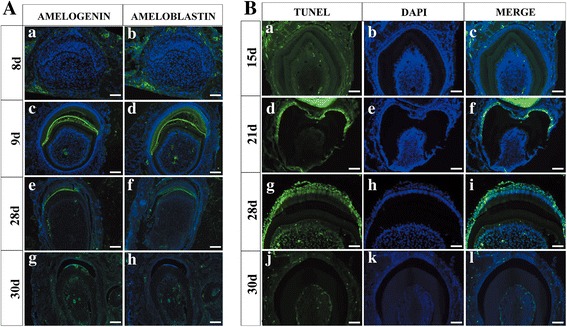
Cytodifferentiation and apoptosis of hKSC-derived dental epithelium in human–mouse chimeric teeth. **A** Immunofluorescence shows expression profiles of amelogenin and ameloblastin in hKSC-derived ameloblasts at various time points in subrenal culture. Amelogenin and ameloblastin expression not detectable until day 9 (a–d), downregulated at day 28 (e, f), and completely silenced at day 30 (g, h), respectively, after subrenal culture. **B** TUNEL assay shows programmed cell death of hKSC-derived ameloblasts at various time points in subrenal culture. Obvious apoptosis signals not detected until day 21 (a–f), reached maximal level at day 28 (g–h), and quenched at day 30 (j–l), respectively, after subrenal culture. Scale bar = 100 μm. 8d 8 days, TUNEL terminal deoxynucleotidyl transferase dUTP nick end labeling, DAPI 4′,6-diamidino-2-phenylindole

Human deciduous teeth begin to develop during the 6th week of gestation, but it is not until the 18th week that the dental epithelium starts to differentiate into enamel-secreting ameloblasts [38]. It takes about 400 days for human deciduous teeth to develop from initiation to eruption [39]. However, in our study, a completely differentiated human–mouse chimeric tooth crown was found within only 4 weeks under the kidney capsule culture (Fig. 2h, i) [22]. Therefore, we sought to examine the developmental process of this rapid differentiation of hKSC-derived dental epithelium to functional ameloblasts and regeneration of human enamel in the chimeric tooth germ. Histological staining indicated that hKSCs aggregated to a well-defined dental epithelial bud at day 5 in subrenal culture (Fig. 4A), corresponding to the bud stage in normal tooth development. This tooth bud underwent typical dental epithelial histogenesis within the tissue recombinant, progressing to the cap stage at day 6 (Fig. 4B) and the bell stage at day 7 (Fig. 4C). The odontoblasts of mouse origin produced dentin at day 8 (Fig. 4D, E) and the hKSC-derived ameloblasts deposited enamel at day 12 (Fig. 4F). Eventually, well-differentiated teeth were formed within 30 days (Fig. 4H). To further confirm the rapid ameloblastic differentiation of hKSCs in chimeric tooth germs, we further performed immunohistochemistry and TUNEL assays to examine the expression profiles of amelogenin and ameloblastin, two molecular markers for differentiating ameloblasts [40, 41], and the programmed cell death at various time points [42]. Strikingly, we found that expression of amelogenin and ameloblastin was not detectable in the hKSC-derived dental epithelia in the recombinants at day 8 (Fig. 5Aa, b), but became strong at day 9 (Fig. 5Ac, d), further reduced to low levels at day 28 (Fig. 5Ae, f), and quenched at day 30 when completely mineralized tooth crowns were formed (Fig. 4I and Fig. 5Ag, h). In addition, TUNEL assay revealed that it was not until 21 days that apparent apoptotic signals could be detected (Fig. 5Ba–f). Apoptotic signals reached the maximal level at day 28 (Fig. 5Bg–i) and were completely quenched at day 30 (Fig. 5Bj–l), when ameloblasts lost their healthy morphology with condensed nuclei in reduced enamel epithelia that underwent degeneration (Fig. 5Ag–h, Bi). These observations provide cogent evidence for the rapid differentiation of hKSC-derived ameloblasts in human–mouse chimeric teeth.

In our previous report, we identified the human origin of hKSC-derived dental epithelial component and the mouse origin of the dental pulp with specific antibodies against human or mouse MHC antigen, respectively, in chimeric teeth to show no contamination of the mouse dental epithelial tissue in the recombinant experiment [22]. In the present study, we further recombined hKSCs with mouse dental mesenchyme genetically labeled with eGFP. Immunofluorescence studies indicated that no GFP-positive cells could be found in hKSC-derived ameloblasts that were marked with SP6 in chimeric teeth (Fig. 4J). In addition, we grafted E13.5 dental mesenchyme with removal of dental epithelium after enzyme treatment into nude mice for subrenal culture for 4 weeks as a further control. All 30 grafted samples either degenerated or formed tiny pieces of bone-like tissues (data not shown). These data provide more evidence to rule out the possibility of mouse dental epithelium contamination in the recombinant experiment.

### Microstructure and mechanical characteristics of regenerated human enamel

**Fig. 6:**
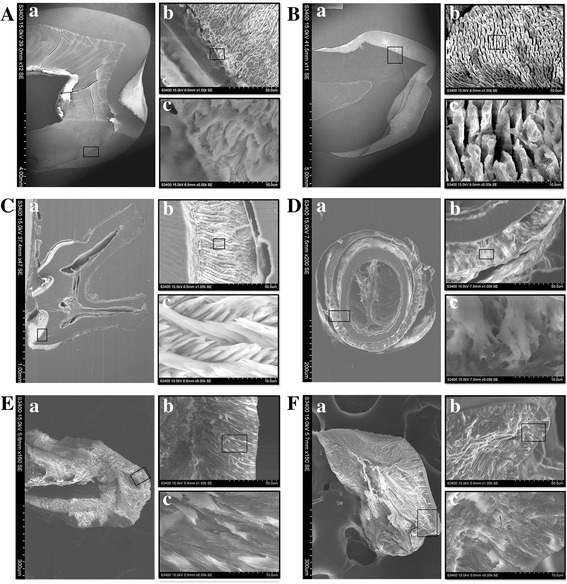
Comparison of microstructure of enamel in human (adult and child), mouse, and human–mouse chimeric teeth by SEM. **A** SEM images of microstructure of adult human tooth. **B** SEM images of child human tooth. **C** SEM images of adult mouse tooth. **D** Cross-section SEM images of human–mouse chimeric tooth after 20 days in subrenal culture. **E** Vertical-section SEM images of human–mouse chimeric tooth grown after 20 days in subrenal culture. **F** SEM images of human–mouse chimeric tooth after 40 days in subrenal culture. Frame regions in (a) and (b) enlarged in (b) and (c), respectively, in each panel

Enamel is the hardest calcified tissue of the body and is structurally distinct from collagen-based calcified tissue. To illustrate whether the rapidly regenerated human enamel is physically and functionally comparable to normal enamel, we examined microstructure and physical characteristics of regenerated human enamel on the surface of human–mouse chimeric tooth utilizing scanning electronic microscopy (SEM) and nanoindentation, respectively. The microscopic characteristic structures of human adult, human child, mouse molar, and human–mouse chimeric teeth after 20 and 60 days in subrenal culture are compared in Fig. 6. The enamel structures of a human adult tooth on macro and micro scales are depicted in Fig. 6A. The enamel prisms were clearly observed in the pattern of complex trajectory (enlarged in Fig. 6Ab, c). Similar results were also observed for the enamel structure of a human child tooth in Fig. 6B. Meanwhile, a more clear running pattern of the enamel prisms arranged in row with alternating orientation was found in the mouse molar (Fig. 6Ca–c). Upon comparison with the microstructure of enamel prism of human and mouse molar, the enamel prism of human–mouse chimeric tooth cultured for 20 and 60 days was orderly aligned as illustrated in Fig. 6D–F, manifesting that the morphologies of enamel prisms of human–mouse chimeric teeth cultured for 20 days (Fig. 6E) and 60 days (Fig. 6F) were almost identical to those of the human molar (Fig. 6A, B). It is well known that enamel is made up of hydroxyl apatite crystals and then arranged in prisms. The appearance of prisms is determined by the orientation of the crystals. According to the study of enamel structure in human molars, the enamel is divided into three layers by the running pattern of the enamel prisms [43, 44]. In this work, similar images of the running pattern of the enamel prisms were observed for the human molar and the human–mouse chimeric teeth cultured for 20 or 60 days, indicating that the enamel prism microstructure of the human–mouse chimeric teeth is likely to grow completely after cultivation for more than 20 days.

**Fig. 7:**
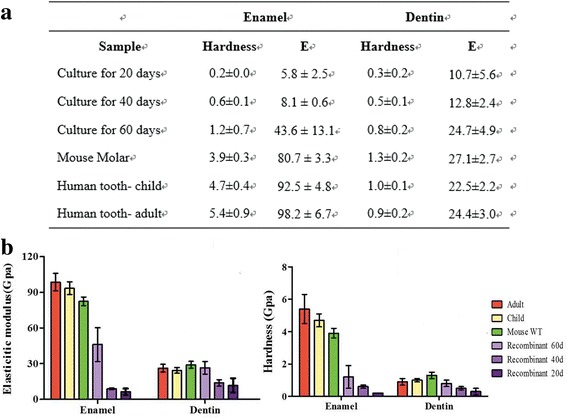
Comparisons of mechanical properties of enamel and dentin in humans (adult and child), mouse, and human–mouse chimeric tooth crown specimens. **a** Statistical data on elastic modulus and hardness of enamel and dentin from nanoindentation tests. **b** Reduced elastic modulus and hardness of enamel and dentin for humans (adult and child), mouse, and human–mouse chimeric tooth crown specimens. *E* elastic modulus, WT wild type, 60d 60 days

As the hardest matrix of the body, enamel is brittle and easily fractioned. It is supported by dentin, an underlying layer of a more resilient calcified matrix, to maintain its integrity and hardness to withstand mechanical force applied during tooth functioning. We therefore tested mechanical characteristics of both enamel and dentin in a whole tooth crown. The average elastic modulus, *E*, and average hardness, *H*, of enamel and dentin regions for human (adult and child), mouse molar, and human–mouse chimeric tooth crown specimens are plotted in Fig. 7. The average *E* and *H* values for the enamel of human–mouse chimeric teeth increased from 6.3 to 46.0 GPa and from 0.2 to 1.2 GPa, respectively, as the culture time increased from 20 to 60 days. Notably, around a 6-fold to 7-fold increase was found for the *E* and *H* of enamel as the culture time increased, which indicates the mineralization effect increases with cultivation time. The average *E* and *H* values for the regenerated enamel in human–mouse chimeric teeth cultivated for 60 days were still 46% and 70% less than those values of the mouse molar. As compared with the mechanical property of mouse molar enamel in the literature [30], very similar results were obtained for the *E* and *H* in this work. The highest values of *E* and *H*, 98.6 and 5.4 GPa, could be found for the enamel of adult human teeth. On the other hand, for the dentin of human–mouse chimeric teeth, the average *E* and *H* values increased from 10.7 to 24.7 GPa and from 0.3 to 0.8 GPa, respectively, as the culture time increased from 20 to 60 days. A 2.7-fold increase was found for the hardness of dentin when the culture time increased from 20 to 60 days, implying its increasing mineralization effect. The average *E* and *H* values for the dentin of human–mouse chimeric teeth cultivated for 60 days reached around 91.1% and 61.5%, respectively, by comparison with those of the mouse molar; the findings were even higher than those of adult human teeth. The mechanical property evaluation of enamel and dentin of tooth-like structures by nanoindentation technique has been reported previously [24]. As compared with those of the mouse and adult teeth, much lower values of hardness and elastic modulus were found for the enamel and dentin of regenerative teeth [24]. A similar tendency was also reflected in this study. Meanwhile, the hardness of the regenerative enamel might be increased by slowdown of tooth development process in culture since the mineralization effect is significantly increased as the culture time increased.

## Discussion

Reciprocal heterotypic recombination of tissues of ectopic origin has been long used as a routine approach to study regulative interactions between tissue components in classical experimental embryology. Mammalian tooth development is dependent upon inductive interactions between epithelium and adjacent mesenchyme [45]. Both epithelial and mesenchymal components in tooth germ are indispensable for tooth development [46, 47]. Sequential and reciprocal interactions between the stomadial epithelium and the cranial neural crest-derived mesenchymal cells regulate tooth morphogenesis and differentiation. Odontogenic potential represents an instructive induction capability of a tissue to induce gene expression in an adjacent tissue and to initiate tooth formation, whereas odontogenic competence indicates the capability of a tissue to respond to odontogenic inducing signals and to support tooth formation. Tissue recombination experiments between isolated mouse molar epithelial and mesenchymal tissues have demonstrated that, during early tooth development, odontogenic potential resides first in the dental epithelium and then shifts to the mesenchyme [48, 49]. At the prebud stages of development (before and at E11.5), the presumptive dental epithelium possesses the potential to induce tooth formation in nondental mesenchyme. In contrast, at the early bud stage (E12.5) the odontogenic potential has switched to the mesenchyme, and this odontogenic mesenchyme is able to instruct nondental epithelium to form tooth-specific structures [48–50]. Our previous report demonstrated that such potential is also conserved in human embryonic dental mesenchymal tissues that are able to induce nondental epithelial tissues, such as human keratinocyte, and able to participate in tooth formation [20]. *In-vitro* bioengineering of primordial tooth germs represents a promising approach for tooth replacement therapy in the future [51]. Either *in-vitro* or *ex-vivo* generation of an implantable biotooth germ should follow the principles of tooth development. Based on this concept, previous studies including ours have demonstrated that human epithelium-derived stem cells, including iPSC-derived epithelium-like tissue, could be induced to participate in tooth formation when confronted with mouse dental mesenchyme with odontogenic potential. However, the efficiency of the induced ameloblastic differentiation of these cells was relatively low, and can be an obstacle for using adult stem cells as an epithelial cell source to develop tooth replacement therapy. In this study, in comparison with our previous report in which around 30% of tooth formation and 10% of ameloblastic differentiation were obtained [22], we achieved 70% and 100% of tooth formation and ameloblastic differentiation, respectively, by application of two key growth factors, FGF8 and SHH, in cell culture and tissue recombination, demonstrating that in the presence of appropriate odontogenic signals an efficient induction of enamel-secreting ameloblasts from hKSCs could be achieved.

FGF8 and SHH have been demonstrated to play pivotal roles in mammalian tooth development. SHH acts as an autonomous signal to regulate dental epithelial cells to proliferate, grow, and differentiate into functional ameloblasts [31, 52]. FGF8 is responsible for the determination of tooth forming sites, induction of several tooth developmental genes, and initiation of tooth development [53]. We did find that, in the present study, application of these two growth factors activated SP6 expression in addition to that of MSX2 and SP3 expression in the hKSC-derived dental epithelium in tissue recombinants. The similar phenotypes in mice lacking the *Msx2*, *Sp3*, or *Sp6* gene, respectively, and their overlapping expression pattern during tooth development raise the possibility that these transcription factors reside or interact closely within a signaling cascade to regulate amelogenesis [35, 36]. Our study indicates that simultaneous activation of these three transcription factors could likely be necessary for initiation of ameloblastic differentiation in hKSCs of the chimeric tooth germ. However, our results also indicate that application of FGF8 and SHH in the cultured hKSCs did not alter the expression of MSX2, SP3, and SP6 but improved stemness of hKSCs. These data strongly suggest that the enhanced efficiency of ameloblastic differentiation in hKSCs is associated with an improvement of cultured hKSC stemness but not their ameloblastic fate.

In the developing human deciduous teeth, ameloblasts begin to differentiate around the 15th week and start to secrete and deposit enamel on the surface of dentin around the 18th week of gestation [38]. It is not until 6 months after birth that ameloblasts undergo apoptosis when the tooth erupts. Of interest, in our study, the differentiation and enamel deposit of hKSC-derived ameloblasts in the chimeric teeth were completed within 4 weeks under subrenal culture and this rapidly generated enamel exhibited a microstructural pattern grossly identical to normally developed enamel. Despite the mechanical property evaluation of enamel structures by a nanoindentation technique revealing much lower values of hardness and elastic modulus for the regenerated enamel than those of adult human and mouse teeth, an increasing tendency for the mineralization effect with cultivation time was discovered in this study. This should be of a significant impact for future clinical practice, since implantable tooth primordia could grow rapidly in the patient oral cavity in a relatively short period of time and further mineralize and mature to attain effective hardness and elastic modulus to withstand mechanical force applied during food chewing.

## Conclusion

We developed a process for efficient induction of enamel-secreting ameloblasts and rapid generation of enamel from hKSCs by treatment of hKSCs with both FGF8 and SHH proteins prior to recombination with mouse embryonic dental mesenchyme. FGF8 and SHH dramatically enhanced stemness of cultured hKSCs. Electron microscopic analysis and a nanoindentation test revealed the formation of intact prisms and an increasing tendency for the mineralization effect with cultivation time in the regenerated enamel. Our results provide an appealing idea for efficient induction of adult stem cells into enamel-secreting ameloblasts.

## Additional file

Additional file 1: **Figure S1.** Showing immunohistochemistry expression patterns of MSX2, SP3, and SP6 in developing human primary tooth germ. **A** MSX2, **B** SP3, and **C** SP6 protein distribution in cap and bell stages of tooth germs: (a, b) 12-week human primary incisor; (c, d) 16-week human primary incisor; (e, f) 19-week human primary incisor. Scale bar = 100 μm (a, c, e), 50 μm (b, d, f). (TIFF 6518 kb)
