# Supplementary figures and images for: Efficient induction of functional ameloblasts from human keratinocyte stem cells
Source: Stem Cell Res Ther. 2018 May 2;9:126. doi: 10.1186/s13287-018-0822-4 (PMC5930762; doi:10.1186/s13287-018-0822-4)

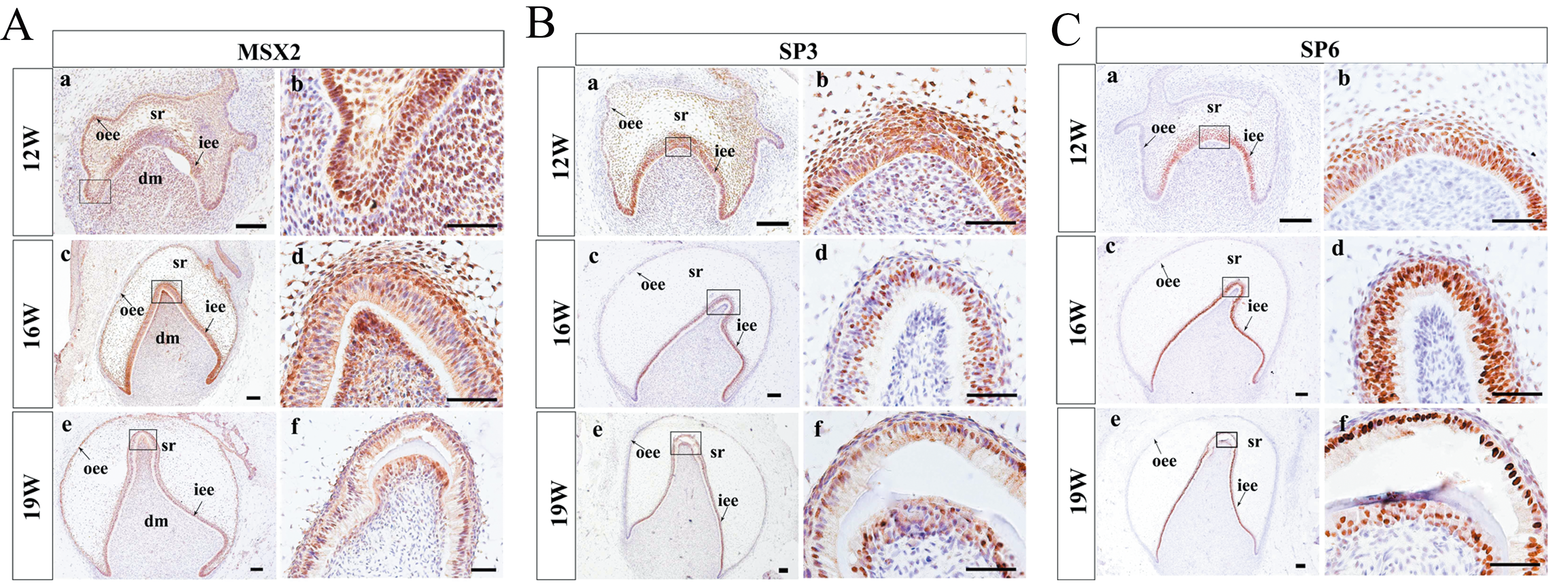

Supplement: Supplementary file 1 — Figure S1. Showing immunohistochemistry expression patterns of MSX2, SP3, and SP6 in developing human primary tooth germ. A MSX2, B SP3, and C SP6 protein distribution in cap and bell stages of tooth germs: (a, b) 12-week human primary incisor; (c, d) 16-week human primary incisor; (e, f) 19-week human primary incisor. Scale bar = 100 μm (a, c, e), 50 μm (b, d, f). (TIFF 6518 kb) [file 13287_2018_822_MOESM1_ESM.tif]
